# Engagement Books

**Published:** 1881-12

**Authors:** 


### EDITORIAL.

### ENGAGEMENT BOOKS.

Enquiries have been made, whether the Editor of the Register will issue "The Dentist's Engagement and Memorandum Book for 1882."

About an hundred copies of the edition for 1881 being on hand, it has been decided not to publish for 1882. The figures in that of '81 can easily and quickly be changed for 1882. They can be had of the dental depots, at perhaps some discount.

